# RNA interference screening demystified

**DOI:** 10.1136/jcp.2008.058735

**Published:** 2009-01-06

**Authors:** C J Lord, S A Martin, A Ashworth

**Affiliations:** The Breakthrough Breast Cancer Research Centre, The Institute of Cancer Research, London, UK

## Abstract

Genetic screens, where the effects of modifying gene function on cell behaviour are assessed in a systematic fashion, have for some time provided useful information to those interested in disease pathogenesis and treatment. Genetic screens exploiting the phenomenon of RNA interference (RNAi) are now becoming commonplace. This article explains the different RNAi screen formats and describes some of the applications of RNAi screening that may be pertinent to the research pathologist.

## RNA INTERFERENCE (RNAI): WHAT, HOW AND WHY?

**Figure 1:**
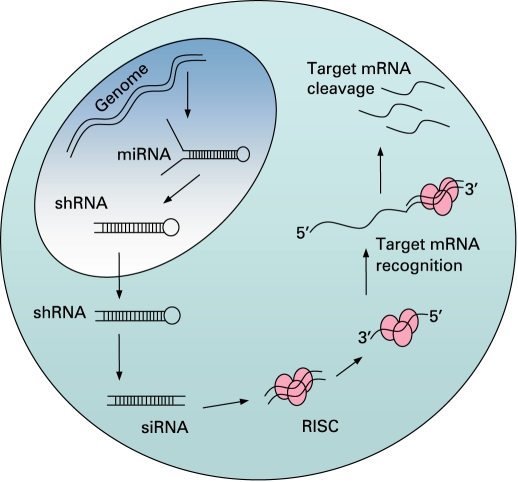
RNA interference. For simplicity we have shown how microRNAs (miRNAs) can mediate RNA interference in mammalian cells by causing the degradation of protein-coding transcripts. Non-protein coding miRNAs are transcribed from the genome and then processed in the nucleus into shorter RNA species bearing a hairpin structure (shRNAs); these hairpin structures, consisting of a “stem” and a “loop”, are caused by base pairing between short regions of the RNA sequence (these form the “stem”) separated by a short sequence that does not form base pairs (which forms the “loop”). shRNAs are exported from the nucleus and further processed into small RNA duplexes (siRNAs) formed from the stem of the shRNA. siRNAs are loaded into the RNA-induced silencing complex (RISC). This complex facilitates binding between one of the siRNA strands and protein-coding mRNAs that have nucleotide sequence complementary to the siRNA. Once siRNA/mRNA binding has occurred, and thus the target mRNA transcript has been recognised, a nuclease in RISC degrades the mRNA, thus ultimately reducing the amount of mRNA that is available for translation and protein production. This mechanism can be exploited experimentally to silence specific genes. Synthetic siRNAs can be delivered into cells by transfection and are readily loaded on to the RISC and mediate degradation of mRNAs with significant sequence complementarity. Viruses or plasmids containing miRNA-coding or shRNA-coding sequences can also be introduced into mammalian cells and these mimic the production of endogenous miRNA and shRNA and are processed into siRNA as before.

Put simply, experimental RNAi allows the research scientist to assess the function of a gene or protein by silencing its expression using synthetic RNAs or plasmids (an effect often referred to as “knockdown”). This technique exploits a physiological mechanism that represses gene expression by causing the degradation of protein-coding messenger RNA (mRNA) transcripts. In mammalian cells, physiological RNAi is primarily mediated by non-protein-coding RNA transcripts, known as microRNAs (miRNAs) (fig 1). miRNAs are produced in much the same way as normal mRNAs, but instead of being translated into proteins, miRNAs are processed into shorter RNA species containing a hairpin structure, known as short-hairpin RNAs (shRNAs). These are in turn processed into short double-stranded pieces of RNA known as short-interfering RNAs (siRNAs). Within a multiprotein complex known as RISC (RNA-induced silencing complex), one strand of a siRNA duplex binds a protein-coding mRNA transcript that bears a complementary nucleotide sequence. This interaction allows a nuclease in RISC to cleave and destroy the protein-coding mRNA, thus silencing the expression of the gene in a relatively sequence-specific manner (fig 1).

In 1998, Mello and Fire demonstrated that potent gene silencing could be experimentally induced by injecting double-stranded (ds)RNA into the nematode *Caenorhabditis elegans*. In this context, dsRNA is processed into siRNA by an enzyme, Dicer.^1^ In mammalian cells, however, the introduction of long dsRNAs induces an interferon response that confounds experimental results, primarily by inducing an interferon response that causes a global shutdown of protein synthesis.^2^ However, synthetic siRNAs, or plasmids expressing shRNAs or miRNAs which are processed into siRNAs, can be introduced into mammalian cells and mediate gene silencing without significant adverse effects.^2^

The experimental use of synthetic siRNAs and shRNA plasmids has profoundly changed the way in which the loss of function of particular genes or proteins is studied. Previously, techniques that were either more time consuming, such as gene targeting (making “knockout” cell lines or animals), or capricious, such as antisense RNA, were used. Alternatively specific inhibitors of a particular protein could be used, but these are limited in scope. Now RNAi reagents can be purchased and trivially used to silence almost any gene in the genome.

As described above, the types of RNAi reagents fall into two main categories, siRNAs and shRNA expression constructs. Table 1 shows the advantages of each reagent format. siRNAs consist of small double-stranded pieces of RNAs (generally over 19 bp), one strand of which matches a complementary sequence in the gene to be knocked down. These siRNAs can be either synthetically synthesised or generated by enzyme digestion of mRNA, a technique known as esiRNA.^3^ The main advantage of siRNAs is ease of use; given their small size, siRNAs are readily transfected into most cell types, and they can be purchased in “ready-to-transfect” format from many commercial suppliers. One limitation of siRNAs, however, is that gene silencing is only achieved over a short period of time; siRNAs are most likely degraded by the host cell and are also not replicated along with the host cell’s DNA. Therefore, as cells proliferate, the proportion of cells carrying siRNA is gradually reduced. To some extent, this short period of silencing (a number of days) has been addressed by chemical modification of siRNA (see www.dharmacon.com), but if long-term silencing is required, shRNA expression constructs are more often the solution. These constructs express miRNAs or shRNAs that are processed by the target cell’s own RNA-processing machinery into siRNA, which in turn mediate gene silencing. These constructs come in a variety of formats and can be purchased either as simple plasmids or as viral constructs that are easier to deliver to a wider range of cell lines. shRNA vector-based reagents can provide long-term, stable gene silencing, as the vectors used are able to integrate into genomic DNA and are thus copied along with cellular DNA. Although the inherent instability of some of the initial viral systems used to express shRNA limited their utility, this problem has now been addressed in the most recent versions that are available from commercial suppliers. One of the more recent advances in shRNA construct design has been the use of vectors that express miRNAs instead of shRNAs. It is proposed that, as this process more closely mirrors the mechanism by which endogenous mammalian miRNAs mediate gene silencing, it leads to an improvement in silencing efficiency.^4^ ^5^ It is also worth noting that these improvements in vector design also raise the possibility of *in vivo* RNAi screens in whole animals, as opposed to *in vitro* screens in cultured cells. Already, individual genes can be targeted in mice using RNAi,^6^ and, in fact, candidate tumour suppressor genes have been validated using this approach.^7^

**Table 1:** RNAi library formats

Library type	Advantages	Disadvantages
siRNA normally arrayed in 96-well or 384-well plates can combine multiple siRNAs targeting different sequences in the same gene in one well (SMARTPools) can be purchased in “ready-to-transfect” aliquots or in larger amounts that require replating can also be arrayed on slides	Consistent quality of reagents Ease of use and readily transfectable Chemical modification of siRNA can limit off-target effects	Finite resource Target cells need to be transfectable Relatively short period of silencing Not suited to pooling strategies
		
shRNA as: plasmid DNA arrayed in 96-well or 384-well plates for transfection viral particles in multi-well plates (one shRNA per well) pools of plasmids or viral particles	Renewable resource Viral vectors enable silencing in difficult-to-transfect cells Suited to pooled screens as well as arrayed screens Stable integration of silencing machinery into host cell genome enables longer-term silencing Varying vector formats available that allow inducible silencing, tracking of silencing machinery, etc	Reduced ease of use: require preparation of plasmid DNA and, in the case of viral-based libraries, viral packaging Viral use often requires biological containment Pooled screens require significant deconvolution

Supplier websites: www.openbiosystems.com; www.dharmacon.com; www.origene.com; www.scbt.com; www.ambion.com; www.sigmaaldrich.com; www.systembio.com.

**Figure 2:**
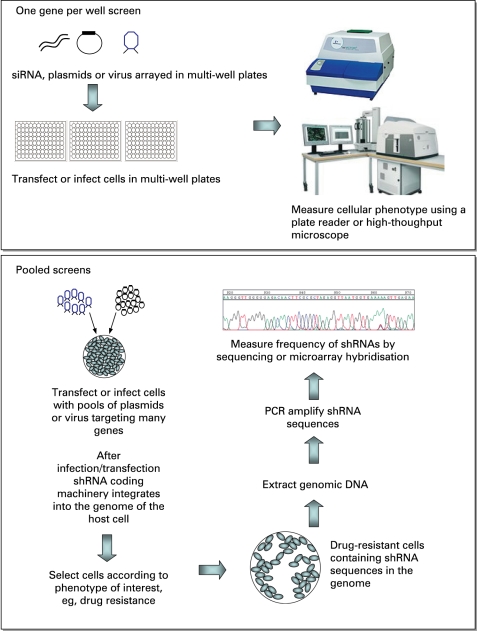
RNAi screening formats. Top panel: One gene per well screens target each gene separately in a multi-well format. Short-interfering RNAs (siRNAs), short-hairpin RNA (shRNA) plasmids or virally packaged shRNA constructs can be used to transfect or infect cells. Various readouts may be used to determine the effect of RNAi on the phenotype of interest; the measurement of cell viability is common, and luminescence-based plate readers are often used for this purpose. Alternatively, high-throughput microscopes may be used to measure cellular phenotypes in screens. Lower panel: In pooled screens, pools of shRNA-expressing vectors are introduced into cells by transfection or infection. Cells are then exposed to a selective agent such as a drug. In this case, shRNAs that cause drug resistance can be identified and quantified by amplifying shRNA sequences from genomic DNA in surviving cells. Microarray analysis is ideal for detecting shRNA sequences, as is Next Generation Sequencing. For more details, see the main text.

siRNAs and shRNA expression constructs are now available as libraries that contain reagents targeting any number of genes. Depending on the application, RNAi reagents targeting a single gene, a particular gene subset, for example protein kinases, or indeed the entire genome may be purchased. The commercial availability of such reagents now means that a number of genes can be screened simultaneously, and relatively straightforwardly, for their involvement in a chosen biological phenotype. The format of how such a screen is performed is largely determined by the choice of RNAi reagent. Both shRNAs and siRNAs are well suited to “one gene per well” screens (fig 2) where cells are transfected or infected in multi-well plates, each well containing RNAi reagents that silence one gene. Normally 96-well or 384-well plates are used for such screens, meaning that the entire genome can be screened using under two hundred 384-well plates or just over six hundred 96-well plates. Obviously such screens are better performed using some form of liquid handling automation, although smaller screens where a gene subset such as the protein kinases are targeted can be performed manually using only ten 96-well plates. The simplest screens of this type use some form of plate reader to measure the effect of RNAi reagents; measuring cell viability by the use of an ATP colorimetric assay is common. However, the recent trend is to measure multiple phenotypes within the same screen, sometimes using high-throughput microscopy, an approach termed “high-content” analysis. For example, Bakal *et al*^8^ recently screened a small gene subset by RNAi using high-throughput microscopy to measure changes in cell morphology. Although the computational requirements to analyse such high-content screens properly are high, these approaches offer the potential to dissect complex biological networks and pathways.

The scale of whole-genome screens that involve the use of hundreds of multi-well plates can often be daunting. Given this, an alternative approach is to use shRNA-expression constructs in “pooled” screens (fig 2). Here, pools of shRNA-expressing vectors are used to infect or transfect cells, which are then selected for the phenotype of interest such as resistance to a small-molecule inhibitor. The target gene in surviving cells is identified by PCR amplification and sequencing of the unique short hairpin sequence of the shRNA vector that encodes the gene-specific shRNA. Relatively new sequencing platforms, such as ABI SOLiD (Roche 454) and Illumina’s Genome Analyzer II are ideal for this purpose as they are able to simultaneously sequence millions of short pieces of DNA, such as PCR products containing short hairpin sequences of shRNAs. An alternative approach for screening deconvolution is to use oligonucleotide microarrays to identify hits from pooled shRNA screens. These technologies can be used to identify shRNA targets that are either selected for (positive selection) or selected against (negative selection). For example, Brummelkamp and colleagues^9^ used a library targeting ∼8000 genes in a positive bar code screen to identify mediators of sensitivity to the anticancer drug Nutlin-3, an inhibitor of Mdm2 that activates the p53 pathway, allowing a more complete understanding of the mechanism of tumour selectivity for this drug.^9^ Such pooled approaches are not limited to drug-sensitisation screens. For example, Green and colleagues screened ∼62 400 shRNAs targeting ∼28 000 mouse genes to analyse the mechanisms that control the cell surface expression of the pro-apoptotic Fas receptor.^10^ The ∼62 400 shRNAs were divided into 10 pools, which were packaged into retrovirus particles and used to stably transduce Fas receptor-negative, K-ras NIH 3T3 cells. Cells in which Fas receptor expression had been induced by RNAi were selected from the total cell population using immunomagnetic beads bearing an anti-Fas antibody. This Fas receptor-positive population was expanded, and the shRNAs that caused expression of the receptor were identified by sequencing shRNAs present in a FasR-positive population. Ultimately this screen identified a mechanism by which the oncogene Ras mediated epigenetic silencing of the Fas gene, thus allowing tumour cells to avoid apoptosis, one of the hallmarks of a cancer cell.

**Glossary**

- RNAi, RNA interference. The process by which RNAs that encode proteins (messenger RNAs (mRNAs)) are degraded in a sequence-specific manner by binding to a short interferring RNA.
- siRNA, short interfering RNA. The RNA species that binds mRNA in the RNA-induced silencing complex (RISC). The mRNA/siRNA interaction activates the nuclease activity of RISC, leading to degradation of the mRNA.
- shRNA, short hairpin RNA. A siRNA precursor, encompassing a “stem” and a “loop” structure formed by base pairing between short regions of the RNA sequence (these form the “stem”) separated by a short sequence that does not form base pairs (which forms the “loop”).
- microRNA (miRNA), a non-protein-coding RNA species. One of the known functions of miRNAs in mammalian cells is in RNA interference
- pooled/non-pooled screens. In non-pooled screens, RNAi reagents targeting one gene are introduced into a single-cell population. Non-pooled screens are normally carried out in multi-well plates, with each well representing RNAi reagents targeting one gene. In pooled screens, RNAi reagents targeting a number of genes are introduced into the cell population at the same time.
- Infection, the process by which DNA, packed within a viral coat, is introduced into cells. DNA-encoding miRNAs or shRNAs can be packaged as virus and infected into cells.
- Transfection, the process by which “naked” or non-virally packaged DNA is introduced into cells. Normally this is achieved by binding DNA to a lipid; the resultant lipid sphere binds to and merges with the target cell’s plasma membrane, releasing the DNA into cells.

## THE PROCESS

The commercial availability and relative ease of use of RNAi libraries now means that most research labs are able to perform an RNAi screen, be it genome wide or targeting a smaller gene subset. Given this, it is worth describing the major issues that should be considered before embarking upon such an enterprise. As with any well thought out experiment, the screen assay to be measured must carefully reflect the clinical, pharmacological or biological question. The assay system must also be robust, reproducible and affordable. Most laboratories use human tumour cell lines for RNAi screens, as these can be routinely grown and transfected but, depending on the phenotype examined, the use of a non-human model system might be considered. The biggest advantage is cost, which can be significantly lower in model organisms because of the ability of *C elegans* and *Drosophila melanogaster* to take up long dsRNAs by feeding or soaking methods, eliminating the requirement for expensive transfection reagents. Recent studies indicate that many results from RNAi screens in model organisms translate to humans.^11^ Of equal importance is the choice of RNAi library. Given the many different formats of RNAi libraries available, this decision is governed by the choice of a pooling or non-pooled approach. If a pooling strategy is chosen, screens have been mostly limited to the vector-based libraries. Whether the assay system requires long-term or short-term gene silencing also needs to be taken into account. Stable integration of vector backbones combined with antibiotic selection cassettes make vector libraries advantageous if long-term silencing is a necessity. However, non-vector siRNA libraries should not be discounted, as transfection efficiencies are normally higher using these smaller nucleic acids, and significant gene silencing can often still be observed after 7 days. Finally, there is the question of reagent lifespan. Vector-based libraries are potentially renewable, but this can be time consuming and expensive. Many prefer the option of having a “ready-to-go” reagent, acknowledging that silencing technology is still improving. Finally, as with any experiment, positive and negative controls should ideally be used in RNAi screens. These controls allow the dynamic range of the assay to be estimated and also, if replicated several times within a library, serve as a form of data quality control. Ideally a negative control would take the form of an RNAi reagent that engages the gene-silencing apparatus but silences a gene that has no effect on the phenotype of interest, such as a synthetic gene in the target cell line such as GFP, the silencing of which can be readily monitored. For example, using cell lines that express a fluorescent protein, such as GFP, in combination with an siRNA that targets GFP, would provide an ideal negative control for many screens. However, the use of such a system may restrict the user to a limited number of cell lines, and the norm is to use non-targeting siRNA.^12^ Although some commercial non-targeting controls are well suited to this purpose, some induce off-target effects (see below) that lead to cell death, and the user is well advised to assess the effect of each control on the phenotype in question, comparing with mock-transfected cells.

Positive controls can be used to define the magnitude of change that is thought to constitute a meaningful biological effect and verify that the screen is returning valid biological data. They also provide biologically relevant thresholds for identifying positive hits from a screen. Comparison of positive and negative controls also allows the screening window coefficient or Z factor^13^ to be estimated, which describes the suitability of the assay system for large-scale screening. Checking that the screen is suitably robust reduces statistical false positives and negatives and saves time and money spent on wasted screen data or attempting to validate false positives.

Once decisions have been made on the factors described above, a typical screening strategy is as follows. Firstly, experimental parameters need to be optimised. Probably, the best place to start is to ensure that high-efficiency transfection or infection can be achieved in the format (multi-well plate or other) that is to be used for the screen. Ideally, one is looking for transfection/infection conditions that enable efficient delivery of the RNAi reagent without causing excessive, non-specific toxicity. One simple trick for assessing RNAi delivery parameters is to transfect or infect cells with RNAi reagents that silence genes that are essential for cell viability and compare cell death with control transfected cells. In human tumour cells, RNAi reagents targeting Polo-like kinase 1 are often used for this purpose, as knocking down this kinase consistently kills most cancer cells.^14^ Different transfection reagents and procedures should be tried here, but more often than not the best place to start is with the standard conditions for most routinely used cell lines found on RNAi reagent suppliers’ websites. Once efficient RNAi delivery can be achieved, positive and negative RNAi reagents should be used to assess whether the screen phenotype can be robustly measured. The key issues here are scale of effect, variability and reproducibility; if the screen assay is such that the difference between positive and negative controls can only be observed rarely, the omens for the screen itself are poor. Having addressed these concerns, one may consider a pilot screen. This is especially important if one is to embark upon a genome-wide screen, and normally involves testing control RNAi reagents alongside a panel of a few hundred selected RNAi reagents. Again this pilot will assess the reproducibility of the positive and negative controls and decipher the hit rate of the screen. If the hit rate is very high, it may suggest lack of specificity of the screen. Pilot screens may also identify problems such as time and space limitations. It is also worth noting here the main reason why screens fail; the inability to precisely reproduce experimental conditions from the initial determination of screen conditions through to the screen itself. Given that most screen conditions are determined empirically (ie, by titrating variables such as cell number, amount of siRNA, etc), it is perhaps wise to standardise all procedures, by using identical batches of cells, media, etc as well as carefully timing and reproducing all of the steps involved in the screen process.

It is also absolutely vital to consider the analysis of screen data before embarking upon the screen itself. Perhaps one of the best methods of screen analysis has been described by Boutros *et al*.^15^ Their CellHTS software package uses a modification of the Z score/median absolute deviation method of defining screen hits. Although we will not go into this form of analysis in detail, it is key that one understands the method of statistical analysis before embarking on a screen, as this will inform how the screen is performed and also allow an assessment of the quality of the screen itself.

Once the screen and data analysis are performed, it is often common to perform a secondary screen using only the control RNAi reagents and those silencing the “hits” identified in the screen. Subsequent to this technical validation, it is necessary to eliminate the possibility of off-target effects, ie, effects that are due to silencing of genes other than the intended targets. Although silencing of genes via RNAi requires high complementarity to the target mRNA, off-target effects may require considerably less, in a similar fashion to the microRNA machinery. A 7–8-nucleotide “seed” region at the 5′ end of the anti-sense strand is most critical in generating off-target effects, particularly when this seed region occurs in the 3′ untranslated region of a potential off-target gene.^16^ At present it is not possible to completely eliminate off-target effects by reagent design alone, although some of the commercial suppliers now sell siRNAs that are chemically modified to limit such effects. However, although some of these chemical modifications are able to reduce off-target effects, they may compromise silencing efficiency. Ultimately, to demonstrate that knockdown of the targeted gene is directly responsible for the phenotype, it is important to perform “rescue or redundancy” experiments.^17^ Rescue may involve reversing the phenotype induced by the siRNA by re-expressing an siRNA-resistant cDNA (one that has mismatches with the siRNA and cannot be targeted for degradation). With the large number of hits that result from RNAi screens, this is often not possible, and, in many cases, overexpression of a cDNA has aberrant effects that make this approach difficult to implement. In most cases, redundancy is sufficient to confirm specificity. This involves demonstrating that multiple RNAi reagents targeting different regions of the same target mRNA cause the same phenotype. Silencing caused by individual RNAi reagents should also be confirmed by quantitative real-time PCR or western blotting the target protein. As alternative approaches, small-molecule inhibitors of the “hit” proteins may be used to show that loss of function can elicit the particular phenotype, as can the use of “dominant-negative” cDNA expression constructs or inhibiting antibodies.

Validating individual hits from a screen can often be a more time-consuming and expensive enterprise than the screen itself. Given this, one may choose to use a more rapid approach that exploits the improvements in bioinformatic analysis that are now available. For example, one can now take the list of “hit” genes from a RNAi screen and subject this list to some form of pathway analysis; where a number of hits from the screen fall into the same biological pathway, or indeed have a well-established physical or function interaction, one has some confidence that the effects are real. To achieve this, one has the option of using many of the pathway analysis tools that are publicly available or even the commercial tools such as Ingenuity (www.ingenuity.com). However, it must be noted that this approach is only as good as the quality of the data in the pathway or interaction database that is used. Furthermore, this approach to validation is perhaps only appropriate for whole-genome RNAi screens, as particular gene subset screens may already be biased towards particular pathways.

**Take-home messages**

- RNAi screens are now becoming part of everyday technology used in the research laboratory.
- There are a number of ways to use this technology based on the type of question asked and the scale of the undertaking.
- RNAi technology has the potential to dissect basic biological phenotypes, as well as providing insight into clinically relevant questions.

## APPLICATIONS FOR THE PATHOLOGIST

As the reader will see below, RNAi screens represent more of a research tool rather than an application that could be used in the diagnostic laboratory. Nevertheless, individual RNAi reagents, rather than RNAi libraries and screens, do serve one obvious application for use in the diagnostic laboratory. The ability of RNAi reagents to knockdown the expression of specific proteins makes them ideal for the validation of antibodies that are to be used in diagnostic tests. For example, cells transfected with siRNAs targeting one gene/protein, when compared with control-transfected cells, serve as ideal controls for identifying the optimal conditions for the use of an antibody.

Where RNAi screens are involved, the interest to the pathologist is their utility in the dissection of basic cellular pathways, but also in more translational areas such as identifying novel therapeutic approaches and mechanisms of drug resistance. To illustrate this, we present a number of “case reports” describing a selection of the more pertinent RNAi screens.

### Case history 1: viral infection

Flaviviruses, such as West Nile virus, represent a significant risk to human health. To understand what determines whether a cell can be infected with West Nile virus, Krishnan and colleagues^18^ performed a genome-wide RNAi screen to identify human genes that control viral internalisation. Using a siRNA library arrayed in 96-well plates, 21 121 human genes were silenced in an immortal cell line and the ability of virus to infect cells was measured by immunostaining cells for expression of a viral envelope protein followed by high-throughput microscopic imaging. This analysis identified 305 host cell proteins that affect West Nile virus infection, including the ubiquitin ligase CBLL1. Bioinformatic analysis of the “hits” identified in the screen also identified a role for proteins involved in endoplasmic reticulum-associated degradation and cell adhesion, not only furthering our basic understanding of how host cells determine viral infection, but also suggesting novel therapeutic approaches.

### Case history 2: myelodysplastic syndrome

5q-syndrome, a subtype of myelodysplastic syndrome, is characterised by a defect in erythroid differentiation and, as the name suggests, is thought to be caused by haploinsufficiency of one gene or a number of genes located within a 1.5-megabase region of chromosome 5q. To identify the causative defects in this syndrome, Ebert and colleagues^19^ used lentivirally expressed shRNAs to target all 40 genes in the 1.5-megabase common deleted region on 5q. This RNAi screen identified that loss of function of the ribosomal subunit protein RPS14 causes a similar phenotype to 5q-syndrome in otherwise normal haematopoietic progenitor cells. Exogenous expression of RPS14 rescued the disease phenotype in patient-derived bone marrow cells.

### Case history 3: drug resistance

Many RNAi screens have been carried out with the aim of exploring the mechanism of resistance to chemotherapeutic drugs. For example, we recently used an RNAi screen to identify novel determinants of response to the commonly used breast cancer therapy, tamoxifen.^20^ Breast tumour cells were transfected with siRNA and then divided into tamoxifen-treated and control groups. Those siRNAs that modified the expected response to tamoxifen were investigated further. This screen showed that silencing of the kinase CDK10 caused resistance to tamoxifen and also other endocrine treatments such as Faslodex. Further investigation showed that CDK10 suppression leads to upregulation of cell signalling pathways that abrogate the effects of tamoxifen, thus causing drug resistance. Notably, patients with breast cancer whose tumours expressed low concentrations of CDK10 were shown to relapse much earlier than those with high CDK10 expression, suggesting that CDK10 may serve as a biomarker. Before this RNAi screen, there was no indication that CDK10 expression could determine response to endocrine treatments, illustrating the benefit of such approaches. Using a similar RNAi screen, Swanton and colleagues^21^ showed that the ceramide-transport protein, COL4A3BP, determined resistance to a number of cytotoxic agents. COL4A3BP expression was also shown to be raised in drug-resistant cell lines and in paclitaxel-resistant ovarian tumours, suggesting that COL4A3BP expression is a cause of drug resistance and a potential target for use in chemotherapy-resistant cancers.^21^ Bernards and colleagues^22^ used a large-scale RNAi screen to discover determinants of trastuzumab (Herceptin) resistance in breast cancer. This screen identified the phosphatidylinositol 3-kinase (PI3K) pathway as modulating sensitivity to the drug, with mutations in a pathway member, *PIK3CA*, conferring resistance to trastuzumab. Significantly, this RNAi screen suggests that analysis of the PI3K pathway may provide biomarkers to predict the likelihood of patient response to trastuzumab treatment.

## SUMMARY

Although RNAi technology is still evolving, it is also now suitably mature that RNAi screens are now becoming part of the everyday armoury of the research laboratory. Hopefully, we have shown you that there are a number of different ways to use this technology, primarily based on the type of question one has and the scale of the undertaking that one wishes to perform. Nevertheless, it is certainly becoming clear that this technology has the potential to allow the pathologist to not only dissect basic biological issues but also questions of a more translational bent.
